# Development and evaluation of the psychometric properties of a digital questionnaire for the self-management of health and well-being in the postpartum period

**DOI:** 10.1186/s12884-023-05899-6

**Published:** 2023-08-25

**Authors:** Paola Bully, Isabel Artieta-Pinedo, Carmen Paz-Pascual, Arturo García-Álvarez, Ema-Q Group, Sonia Alvarez, Pilar Amorrortu, Mónica Blas, Inés Cabeza, Itziar Estalella, Ana Cristina Fernández, Gloria Gutiérrez de Terán-Moreno, Kata Legarra, Gorane Lozano, Amaia Maquibar, David Moreno-López, Mª Jesús Mulas, Covadonga Pérez, Angela Rodríguez, Mercedes Sáenz de Santamaría, Jesús Sánchez, Gema Villanueva, Maite Espinosa

**Affiliations:** 1 University of the Basque Country, Barrio Sarriena, S/N, 48940 Leioa, Spain; 2 Paola Bully Methodological and Statistical Consultant, C/ Barrio La Sota, Sopuerta, 48190 Spain; 3 Osakidetza-Basque Health Service, Biocruces-Bizkaia Health Research Institute, C/ Edificio Biocruces 3. Plaza De Cruces, 48903 Barakaldo, Spain; 4 Primary Care Midwife Zuazo Health Centre, Osi Barakaldo-Sestao-Osakidetza, C/ Lurkizaga Kalea, S/N, 48902 Barakaldo, Spain; 5 School of Nursing, University of the Basque Country, C/ Barrio Sarriena S/N, Leioa, 48940 Spain; 6 Midwifery Training Unit of the Basque Country, Hospital de Basurto-Osakidetza, C/ Montevideo Etorbidea 18, Bilbao, 48013 Spain; 7 Primary Care Midwife Markonzaga Health Centre, OSI Barakaldo-Sestao-Osakidetza, C/ Antonio Trueba Kalea, 17, Sestao, 48910 Spain

**Keywords:** Puerperium, Biopsychosocial needs, Web tool screening, Psychometric properties

## Abstract

**Background:**

Despite the fact that the Global Strategy for Women’s, Children’s and Adolescents’ Health (2016–2030) recognises the special importance of care for women during the postpartum period, thus highlighting the need to identify and measure any condition that may affect the welfare of pregnant women in any way, this is one of the most neglected stages in the health system. Given the absence in our area of global, efficient instruments, the objective of this study was to design a complete, specific measurement tool with good metric qualities in digital format for the evaluation of self-reported health and well-being during the puerperium, to conform to what was proposed by the ICHOM.

**Methods:**

A cross-sectional study was carried out to evaluate the psychometric characteristics of a digital measurement tool. The development of the tool was carried out in 4 steps, following the recommendations of the International Test Commission. It was tested on 280 puerperas attending primary healthcare appointments in the Basque Healthcare System (Osakidetza), and they did the newly created survey, answering all the questions that had been selected as the gold standard. The average age of the women was 34.93 (SD = 4.80). The analysis of the psychometric characteristics was based on mixed procedures of expert judgment (a focus group of healthcare professionals, an item evaluation questionnaire and interviews with users) and quantitative evaluations (EFA, CFA, and correlation with gold standard, ordinal alpha and McDonald’s omega).

**Results:**

The final version of the tool comprised 99 items that evaluate functional state, incontinence, sexuality, breastfeeding, adaptation to the role of mother and mental health, and all of these questions can be used globally or partially. It was found that the scores were valid and reliable, which gives metric guarantees for using the tool in our area.

**Conclusions:**

The use of this comprehensive concise tool with good psychometric properties will allow women to take stock of their situation, assess if they have the necessary resources, in psychological and social terms, and work together with midwives and other healthcare professionals on the most deficient areas.

## Introduction

Postpartum care has been described as the most neglected aspect of maternity services and generally, fewer resources are devoted to it than to prenatal care or delivery [1–3]. In our health system, if there is no particular condition that requires special attention, visits to the midwife are usually limited to two, one in the first 10 days after the birth and the other a month after that [4]. Consequently, attention to health from a comprehensive perspective and centered on the needs of women, which includes the promotion of health and its follow-up, is impeded. This happens despite the fact that it is a crucial stage, since the health of the baby and even the rest of the family will depend on the current and future health of the mother, in the short and long term [3].

The main objectives of care for women in the postpartum period are to identify risks, problems and complications, promote the health of the mother and the newborn, and support parents in the new situation of having a baby [5]. Along with the available resources, Maternal Education (ME) focuses on addressing some of these objectives, especially those related to the promotion of health and preparation for motherhood. However, until now and at least in our area, matters related to postpartum are treated at an inappropriate time for women in ME sessions, between weeks 32 and 42, which means that the information is almost ignored. Possibly due to this, one of the demands of the women themselves [6, 7] is to adapt ME to the needs of this period, giving it greater continuity and providing relevant information at each stage. Puerperal in our area also want the health system to allow them to have the autonomy to manage their own health and that of their babies [8, 9].

Likewise, the Global Strategy for Women’s, Children’s and Adolescents’ Health (2016–2030) recognises the special importance of care for women during the postpartum period, highlighting the need to identify and measure not only serious morbidity, but also any another condition that may affect women’s well-being in some way or prevent them from progressing in their recovery and adapting to motherhood [10]. They also recommend follow-up tools with solid data that will serve as a basis for decision-making and greater transparency and accountability in the health system as a way to transform services provided at this stage. Consequently, it is essential to have valid, reliable instruments that will enable the health system to gather information as a basis for improving and monitoring postpartum services, but which will also enable women to assess their own health and seek resources to meet their needs [10].

In line with the above, the International Consortium for Health Outcomes Measurement (ICHOM) carried out a study aimed at defining a minimum set of appropriate outcome measures at international level to evaluate and improve perinatal care, including a postpartum period up to six months [11]. The outcome measures proposed as the most important according to the ICHOM are survival, morbidity, patient satisfaction with care, and health and well-being reported by the patients themselves. Among the health and well-being results reported by patients, the areas classified as most important for women are (1) health-related quality of life (HRQOL), (2) incontinence, (3) pain in sexual relations, (4) confidence and success in breastfeeding, (5) transition and adaptation to the role of mother, and (6) mental health [11].

It is indisputable that there are large numbers of tools available for the postpartum period, aimed at identifying signs and symptoms of a physical and psychological nature. There are instruments – self-administered or requiring administration by a professional – to identify aspects such as depression and/or anxiety [12–16], evaluate urinary incontinence [17], efficacy with breastfeeding [18–20], quality of life [21–24], fatigue [25], quality of sleep [26], quality of sexual relations [27], social support [28], and the mother–child bond [29, 30].

However, there are no well-validated self-report instruments that give comprehensive, efficient coverage of the 6 areas identified by the ICHOM as being important for women. This means that the only possible way to measure the results reported by patients in the postpartum period, covering the 6 areas, is to use a combination of tools [31, 32], as the ICHOM itself suggests. This means carrying out a fragmentary evaluation and analysis of women's health, which is not the ideal method for approaching health from a thorough, person-centred perspective. It is not a useful basis for providing comprehensive family and community healthcare (such as consensual care plans, care management, distance monitoring or searches for social resources and support networks); nor does it help women themselves to manage their own health.

Given the absence in our area of global, efficient instruments, the objective of this study was to design a complete, specific measurement tool with good metric qualities in digital format for the evaluation of self-reported health and well-being during the puerperium, to conform to what was proposed by the ICHOM. This instrument or dossier of scales that have optimized the various instruments that measure these issues separately can be used globally or partially, making use of 1 or more sections separately. It is housed in the *EMAeHealth app*, which we have created using a collaborative research process [33] in which women, healthcare professionals, managers and researchers have participated. EMAeHealth has, among other things, an area for self-management of health, which enables women to evaluate their own needs, as a basis for making informed and/or shared decisions regarding their health and that of their families. This digital tool was designed as a complement to Maternal Education (ME), with resources that facilitate its accessibility, continuity and adaptation to the health needs of each woman.

This article describes how the instrument was developed to assess the priority health and well-being needs of women in the postpartum period, and analyzes its psychometric properties.

## Method

### Design

This study is part of a larger body of research in which the perceptions and needs of women during pregnancy, childbirth and postpartum were analysed, as well as the resources available to them for adapting to each moment of the process. The protocol is now available for consultation [34].

It is a cross-sectional study designed to evaluate of the metric characteristics of a digital tool for detecting health needs in the postpartum, and it was carried out between September 2019 and June 2022 in the Basque Health Service (Osakidetza). This is a public health service that serves a population of just over two million inhabitants and that currently has 7 hospitals where women give birth. Each hospital coordinates with a set of primary healthcare centres for pregnancy and postpartum follow-up.

### Procedure

The postpartum health needs detection questionnaire was created in four steps:

1. Focused review of the scientific literature.

Two bibliographical searches were carried out of English and Spanish databases: PubMed, Web of Science, Embase, CINAHL, PsychLIT, PsycINFO, PsicoDoc, IBECS, Cochrane library plus and Google Scholar.

In the first search, articles related to postpartum health needs were looked for with the aim of identifying relevant constructs and defining them. The search terms we decided to use were those referring to psychosocial and health factors of priority for women (functional status, depression, anxiety, social support, breastfeeding, sexual activity, parental auoefficacy, etc.), as well as those relating to the specific period (postpartum, post-delivery, postnatal, puerperium…) both in free text and in controlled language. This work was checked by a group of midwives belonging to the research team, and they evaluated its fit for the area where the study was being carried out.

The second search looked for instruments that measured the previously identified constructs, with a double aim: 1) to operationalize them by generating a pool of items and 2) to choose the gold standard. To the terms used in the first search were added those referring to tools (tool, instrument, questionnaire, survey, test, data collection, measure…) and their metric properties (validity, reliability, psychometric, etc.). The “consensus-based standards for the selection of health measurement instruments (COSMIN)” checklist was used to evaluate the quality of the metric properties of the existing tools. This checklist describes the validity (content, construct and criteria), reliability (internal consistency, reproducibility, measurement error), and responsiveness of a questionnaire [35].

2) Review of the constructs and items by a committee of experts.

A multidisciplinary team was formed, with 4 primary care midwives, 3 puerperal and paediatric nurses, 1 paediatrician, 3 psychologists, 3 methodologists (a psychometrist and 2 researchers in health sciences) and 2 puerperal women. In order to avoid bias, the purpose of the tool to be developed and the definition of the aspects to be evaluated were explained in writing. Each of the 192 items from the initial pool was evaluated individually for (A) its relevance to a positive postpartum experience and (B) its fit to the reference population. They also checked (C) the clarity of the items and (D) the relevance of the response scales, giving them a score of 0 to 10 in each aspect. An average was estimated for each item and those that did not obtain a score of 8 or higher in relevance and fit to the population were eliminated. Any questions that were still considered important and suitable, but did not obtain averages equal to or higher than 9 points in clarity and relevance on the answer scale were reformulated. Finally, using an open question, the experts assessed the need to include new questions or answer categories. The resulting questionnaire was piloted with a sample of 12 women, who reported on their perception of the relevance, fit and clarity of each of the items.

3) Preliminary analysis of the properties of the instrument.

In this phase, the web layout of the pilot questionnaire was created and the gold standards selected for each construct were administered to a sample of 100 puerperas.

The women were recruited by their midwife in postpartum check-ups. They were offered the option of receiving a link to the questionnaire in digital format. They were also encouraged to share the link with other women in the same situation. All pregnant women over 18 who spoke enough Spanish to understand and answer the questions presented could be included. When the woman accessed the link, she received information about the characteristics of the study, the type of use that would be made of the data (for research purposes only) and about the possibility of withdrawing from the study at any time without this compromising her standard of care. The questionnaire was only filled in if informed consent was given.

Once the information was gathered, an analysis was carried out to evaluate the presence and patterns of absent results, atypical results and compliance with the basic assumptions underlying the general linear model (GLM). Next, the descriptive statistics of each item were calculated (% cases chosen in each option, mean, standard deviation, asymmetry and kurtosis). For the analysis of the internal structure, decision-making techniques were used for the optimal number of factors to be extracted within each construct, and exploratory factorial analyses (EFA) were carried out. The internal consistency of each section was also calculated, as well as how much this indicator would vary if each item were removed. If items had low levels of inclusion factor, and their removal increased the internal consistency of the section, they were eliminated.

4) Management and analysis of the metric properties of the final version.

The findings in the pilot (high commonalities, no cross-loadings, strong primary loadings per factor, high number of indicators per factor and no missing results), and the moderate length of each section of the questionnaire (15 items maximum), suggest that a sample size over 200 offers sufficient statistical power for the CFA of data [36, 37]. In addition, the possible effect of other variables was considered, such as age, parity, nationality (Spanish/immigrant), level of education (low/medium/high) or paid employment (yes/no). Following the same procedure for gathering information as in the pilot, registered healthcare professionals asked 443 women to take part in the study, while another 64 were added by other healthcare professionals or through informal contact between participants.

A preliminary analysis of the information gathered was carried out in order to refine the data and check compliance with the basic assumptions of the GLM. After that, the fit of the models resulting from the EFAs of step 3 was tested through confirmatory factor analysis (CFA). Given the ordinal nature of the items, the estimation method used was diagonally weighted least squares (DWLS) using a polychoric correlation matrix. The evaluation of fit of the model to the data was based on the value of the chi-square/df ratio, together with information provided by the comparative fit index (CFI), the root mean square error of approximation (RMSEA) and its standardization (SRMR). Models with chi-square/df ratio results less than 5, equal to or greater than 0.90 in CFI and equal to or less than 0.10 in RMSEA and SRMR were considered acceptable [38, 39]. The pattern of correlation with other variables to obtain evidence of external convergence was analysed using Spearman’s rank correlation coefficient (rs). Finally, the analysis of the internal consistency of the sections was carried out using the coefficients ordinal alpha (ordinal α) and McDonald’s omega (ω). As a criterion to determine the presence of a possible source of distress, we propose using scores above the 75th percentile value for risk factors and below the 25th percentile for protective factors. The statistical program R (v.4.0.2) was used.

## Results

The main results of the four phases completed before reaching the optimized version of the questionnaire are described below:

### Phase 1. Focused search of the scientific literature

After the first search, it was established that, in addition to the characteristics and evolution of the birth (newborn weight and state of health, type of delivery and surgical procedures performed, e.g. episiotomy), the variables that best determine well-being during the postpartum period could be classified into the 6 groups proposed by the ICHOM. These comprised: (1) functional status/quality of life related to health: possible complications (e.g. haemorrhages, diarrhoea or fever) pain and ability to perform daily activities, all of which are aspects linked to physical recovery in the puerperium [3, 40, 41]. (2) Incontinence as a sequela in the late postpartum period [41, 42]. (3) Recovery of sexual activity and satisfaction [43, 44]. (4) Successful breastfeeding [18]. (5) Transition and adaptation to the role of mother: evolution/confidence in parenting and the satisfaction of the newborn’s needs [45]. (6) Mental health: aspects related to body dissatisfaction, lack of sleep [46] and moods/postnatal depression [41, 47].

The second search focused on tools for the evaluation of these aspects. As a result of this process, it was concluded that there was a need to create a new, complete, updated tool that would be suitable for our social and health environment online.

Table 1 presents the sections that were established as most relevant, and the first column shows the number of items that were initially generated to operationalize each section.

**Table 1:** Evolution of the number and distribution of the items

	**Nº Items**				
**Sections**	Initial	After experts’ opinions	After pilot with 12 women	After analysis with 100 women	Final
1) Functional state					
1.1 Alarm signals	9	11	12	12	12
1.2 Pain	7	7	8	8	8
1.3 Functionality/QOL	5	4	4	4	4
2) Incontinence	6	1	1	1	1
3) Sexuality					
3.1Activity/satisfaction	35	14	15	15	11
3.2 Contraception	3	3	3	3	1
4) Breastfeeding					
4.1 Knowledge	9	9	9	9	6
4.2. Practice	20	15	15	15	13
4.2 Self-confidence	30	6	6	6	6
5) Adaptation to role of mother					
5.1Parental self-efficacy	20	11	11	11	11
5.2 Perceived social support	12	9	9	9	6
6) Mental health					
6.1 Self-image	10	7	8	8	8
6.2 Sleep problems	14	6	7	7	6
6.3 Depression	7	7	7	7	6
**Total**	194	117	115	115	99

In addition, the questionnaires that would be used as gold standards were chosen to determine the convergent validity of the instrument. These, together with their psychometric properties in our sample, are presented in Table 2:

### Phase 2. Review of the constructs and items by a committee of experts

**Table 2:** Gold standards and their psychometric properties in our sample

		**Fit of the original theoretical model**				
**Sections**	**Nº items**	**χ2; p; χ2/df**	**CFI**	**TLI**	**RMSEA (90% CI)**	**SRMR**
1) Functional state						
SF-12 v2 [48]	12	251.46; < .01; 4.83	.98	.97	.11 (.10-.13)	.10
2) Incontinence						
ICIQ [49]	4	2.21; .33; 1.10	.99	.99	.04 (.00-.32)	.03
3) Sexuality						
SFQ [50]	14	49.36; < .01; 1.90	.97	.96	.10 (.06–0.16)	.10
4) Breastfeeding						
PBSES [51]	20	181.79; < .01; 3.56	.99	.98	.10 (.09-.12)	.10
5) Adaptation to role of mother						
PSOC [52]	17	334.06; < .01; 2.85	.98	.97	.08 (.07-.09)	.07
MOS-SSS [53]	20	495.55; < .01; 3.26	.99	.99	.09 (.08-.10)	.05
6) Mental health						
EDI [54]	10	143.51; < .01; 4.48	.99	.99	.11 (.09-.13)	.07
ISI [55]	7	369.75; < .01; 2.46	.99	.97	.09 (.00-.19)	.09
EPDS [56]	10	116.78; < .01; 3.43	.99	.98	.09 (.07-.11)	.10

*ne* not estimable

After the review by the committee, the 81 items that obtained a median score of less than 8 in “Relevance” were eliminated, leaving almost all the scales with a lower number of items than at the beginning (see Table 1, column 2) and 2 new items were added to the questions on alarm signals. Finally, the content of 5 items was reformulated or qualified to increase their clarity and the number of possible responses to each item was homogenised, so that they were all a Likert scale with five alternatives.

After this version was given to the group of 12 women, a key question was added to the scales of pain, incontinence, sexual activity, practice and self-efficacy with breastfeeding, sleep problems and emotional difficulties/depression, so that the respondents would not have to answer all the questions if they did not have that problem. Additionally, the research team carried out a second check of the eliminated items, in case they considered it necessary to reinstate any questions, and the decisions made previously were reaffirmed.

### Phase 3: Preliminary analysis of the properties of the instrument

To evaluate the comprehensibility, readability, duration and initial properties of the final questionnaire (comprising 62.75% of the initial items), it was formatted and a pilot was carried out with 100 postpartum women.

The results showed that it was likely to be used, since it takes around 25–30 min to complete it in its entirety and much less if the scales are used separately. Moreover, most of the respondents considered it easy to understand and interesting.

Based on the findings of the exploratory and internal consistency factor analyses, it was decided that all the elements would be kept, pending further evidence regarding their function.

### Phase 4: Management and analysis of the metric properties of the refined version

#### Characteristics of the participants

Of the 443 women invited to participate by the research team, 348 women answered at least one question, and 216 (48.75%) completed the entire questionnaire. A further 64 were completed by puerperal women contacted through other healthcare workers or through informal contact between participants. Finally, 280 puerperal women with a mean age of 34.93 (SD = 4.74) gave their consent and answered all the questions. The characteristics of the participants can be seen in Table 3.

**Table 3:** Characteristics of the participants

	**n (%)**
**Age**	
< 30	20 (7.1)
30–34	92 (32.9)
35–39	103 (36.8)
> = 40	38 (13.6)
**Nationality**	
Spanish	253 (90.4)
Foreign	27 (9.6)
**Educational level**	
Without schooling/Primary	5 (1.8)
Secondary/Vocational training	16 (5.5)
Sixth form/Further Education	91 (32.5)
University degree	175 (62.3)
**Paid employment**	
Yes	228 (81.4)
No	52 (18.6)
**Type of birth**	
Normal vaginal	179 (63.9)
Vaginal assisted with suction cups, forceps or spatulas	53 (18.9)
Breech	1 (0.4)
Caesarean	45 (16.1)
Other	2 (0.7)
**Nº babies born in this delivery**	
1	273 (97.5)
2	7 (2.5)
**Parity**	
Primiparous	184 (65.7)
2 or more	96 (34.3)
**Epidural anaesthesia during birth**	
Yes	250 (89.3)
No	30 (10.7)
**Episiotomy**	
Yes	82 (29.3)
No	198 (70.7)
**Tearing during the birth**	
Yes	136 (48.6)
No	144 (51.4)
**Complications during the birth**	
No	193 (68.9)
Yes, fever	39 (13.9)
Yes, high blood pressure	6 (2.1)
Yes, postpartum haemorrhage that required transfusion, and/or ICU admission	2 (0.7)
Yes, manual placenta extraction	8 (2.9)
Yes, other complications	32 (11.4)
**dDays since delivery, M (SD)**	45.3 (16.8)

After checking for the absence of patterns of missing results and impossible results in the data, we proceeded to make a formal description of each item (Arithmetic Mean (M) and its 95% confidence interval (95%CI), standard deviation (SD), asymmetry index (AI) and kurtosis index (Ku.)), and evaluate the internal structure, reliability and convergent validity of each of the sections that make up the questionnaire.

##### Functional state

###### Alarm signals

 This is a one-dimensional scale (χ^2^ 2 = 30.96, df = 27, *p* = 0.273, χ^2^/df = 1.15, CFI = 0.96, TLI = 0.95, RMSEA (90% CI) = 0.02 (0.00-. 05), SRMR = 0.12) made up of 11 binary items (see Table 4). Its internal consistency is around what was expected, given the heterogeneity of its content (ordinal α = 0.67; ω = 0.47).

**Table 4:** Characteristics of the items that measure alarm signals in the postpartum period

	Min–max	M	SD	AI	Ku	λ_ij_
Vaginal bleeding more abundant than a period	0–1	0.05	0.22	4.14	15.28	.24
Vaginal discharge/secretion with bad smell	0–1	0.06	0.23	3.83	12.75	.36
Temperature higher than 38ºC (fever) in the last 12 h	0–1	0.00	0.00			.ne
Temperature higher than 38ºC (fever) now	0–1	0.00	0.00			.ne
Feelings of extreme tiredness	0–1	0.20	0.40	1.47	0.18	.46
Feelings of dizziness or faintness sometimes after the birth	0–1	0.23	0.42	1.29	-0.33	.38
One or both breasts hard or swollen even after breastfeeding	0–1	0.15	0.35	2.01	2.03	.77
Red marks on the breast	0–1	0.08	0.27	3.14	7.93	.60
Cracks in one or both nipples	0–1	0.22	0.42	1.34	-0.20	.59
Difficulties in seeing, flashes or other sudden changes in vision	0–1	0.08	0.28	3.05	7.37	.43
Constipation	0–1	0.45	0.50	0.20	-1.98	.23

λ_ij_ saturation or factor loading, *ne* not estimable

Positive response to any of the items is indicative of potential problems that may require specialized attention.

###### Pain

 This is a scale made up of 8 items: 1 binary that acts as a key question; and 7 politomic ones to locate the origin and intensity of the pain (see Table 5). These 7 elements are adjusted to both bidimensional models (χ^2^ = 14.49, df = 13, *p* = 0.340, χ^2^/df = 1.11, CFI = 0.96, TLI = 0.94, RMSEA (90% CI) = 0.03 (. 00-0.09), SRMR = 0.07). 3 belong to the section on specific postpartum pain and 4 to more non-specific or general pain. The internal consistency of the subsection on specific pain is good (ordinal α = 0.73; ω = 0.71) while that of nonspecific pain is lower, as expected for its content (ordinal α = 0.51; ω = 0.50).

**Table 5:** Characteristics of items that measure pain in specific areas of the body

	Min–max	M	SD	AI	Ku	λ_ij_
Do you have pain in any part of your body?	0–1	0.44	0.50	0.25	-1.95	na
The genital and/or anal area	0–4	0.90	0.96	1.06	0.83	.34
One or both breasts	0–4	0.67	0.97	1.54	2.12	.84
One or both nipples	0–4	0.85	1.11	1.22	0.60	.81
The abdominal area (stomach)	0–4	0.61	0.79	1.01	0.05	.17
Head	0–4	0.50	0.89	1.88	2.89	.59
One leg, accompanied by local swelling, heat and redness	0–4	0.12	0.44	4.24	20.19	.70
Other parts of the body	0–4	0.70	1.04	1.48	1.42	.32

λ_ij_ saturation or factor loading, *na* not applicable

Scores of 3 or higher on the sum of specific pain or general pain items are considered high.

###### Functionality/QOL

 This is a one-dimension scale (χ^2^ = 8.76, df = 2, *p* = 0.003, χ^2^/df = 4.38, CFI = 0.92, TLI = 0.91, RMSEA (90% CI) = 0.01 (0.00-0.12), SRMR = 0.02) made up of 4 items (see Table 6), with high internal consistency (ordinal α = 0.82; ω = 0.86) and high correlation with the scores obtained in the SF-12 v2, used as the gold standard (rs = 0.71).

**Table 6:** Characteristics of the items that measure functionality

During the last 4 weeks …	Min–max	M	SD	AI	Ku	λ_ij_
My physical condition has limited me in performing basic baby-care tasks (e.g. breastfeeding, bathing, changing diapers.)	1–5	1.81	1.11	1.29	0.82	.80
My physical condition has limited me in moderate efforts such as moving a table, sweeping or mopping the house or walking for more than an hour	1–5	2.13	1.20	0.74	-0.46	.93
To what extent has pain made it difficult to carry out your usual activities?	1–5	1.91	1.03	1.12	0.52	.79
How often has your state of health made it difficult to carry out social activities (such as visiting friends or family)?	1–5	2.00	1.02	0.66	-0.36	.59

λ_ij_ saturation or factor loading

Scores of 12 or higher on this functionality scale are considered indicative of difficulties.

##### Incontinence

This is a very simple scale, made up of one single item; its formal description is shown in Table 7. This item has a high correlation with the scores obtained in the ICIQ (rs = 0.94). It is unnecessary to calculate the internal consistency or the factor loading.

**Table 7:** Characteristics of the item that measures incontinence

	Min–max	M	SD	AI	Ku	λ_ij_
Do you have leakage of urine, gas or faeces?	0–1	0.23	0.42	1.32	-0.26	na

λ_ij_ saturation or factor loading, *na* not applicable

The presence of incontinence is considered an aspect that may impair the quality of life and therefore require specific attention.

##### Sexuality

This is a scale made up of 12 items: 1 binary that acts as a key question and is used to indicate whether the woman has resumed sexual activity; 10 politomic questions, of which 6 are used to find out the degree of satisfaction with sexuality; and 4 questions to locate the possible origin of sexual discomfort, plus 1 additional question to evaluate knowledge about contraception (see Table 8). The 10 elements that measure sexual satisfaction fit the proposed bidimensional model very well (χ^2^ = 34.80, df = 33, *p* = 0.382, χ^2^/df = 1.11, CFI = 0.99, TLI = 0.99, RMSEA (90%CI) = 0.03 (0.00-0.09), SRMR = 0.06). The internal consistency of both the subsection of satisfaction (ordinal α = 0.93; ω = 0.91) and of pain (ordinal α = 0.89; ω = 0.87) are very good. The link of the scores with the gold standard in the pain subsection was moderate and inverse (rs = -0.36) and with the satisfaction subsection it was high and positive (rs = 0.76).

**Table 8:** Characteristics of the items that measure sexuality

	Min–max	M	SD	AI	Ku	λ_ij_
Have you resumed sexual activity (alone or as a couple)?	0–1	0.30	0.46	-0.90	-1.19	na
I have felt pain or discomfort……						
When touching or stroking the vulva and the perineum, during sexual stimulation	1–5	2.69	1.13	0.33	-0.33	.69
At the moment of vaginal penetration	1–5	2.66	1.18	0.25	-0.65	.95
During vaginal penetration	1–5	1.82	1.10	1.23	0.61	.96
When vaginal penetration is over	1–5	3.10	2.30	0.64	-0.82	.78
During the last 4 weeks, rate your degree of satisfaction with…						
Sexual desire or interest	1–5	3.58	1.15	-0.77	-0.05	.78
Intensity of sexual excitement	1–5	3.27	1.11	-0.21	-0.78	.93
Quality of your orgasms	1–5	3.25	1.10	-0.15	-0.99	.82
Disinhibition and surrender to sexual pleasure during sexual intercourse	1–5	3.08	1.07	0.10	-0.96	.78
Your concentration during sexual activity	1–5	4.29	0.92	-1.34	1.61	.84
Ease of lubrication during sexual activity	1–5	2.82	1.49	0.09	-1.43	.81
I know all the options available to avoid pregnancy at this moment	1–5	1.70	0.46	-0.90	-1.19	na

λ_ij_ saturation or factor loading, *na* not applicable

Scores of 15 or less are considered indicative of dissatisfaction with sexual activity.

##### Breastfeeding

This is a section made up of 25 items, divided into the subsections Knowledge, Practice and Self-confidence in breastfeeding.

###### Knowledge about Breastfeeding

This is a one-dimensional scale (χ^2^ = 17.53, df = 9, *p* = 0.042, χ^2^/df = 1.95, CFI = 0.99, TLI = 0.99, RMSEA (90%CI) = 0.08 (0.02-0.13), SRMR = 0.06) made up of 6 politomic questions with 5 response alternatives that evaluate some beliefs regarding breastfeeding (see Table 9). The internal consistency was high (ordinal α = 0.89; ω = 0.83). The correlation with the PBSES scores was moderate (*r* = 0.30).

**Table 9:** Characteristics of the items linked to breastfeeding

	Min–max	M	SD	AI	Ku	λ_ij_
The benefits of mother's milk are long-lasting, even after the baby has been weaned	1–5	4.33	0.87	-1.27	1.52	.78
Breastfeeding increases the mother–child bond	1–5	4.37	0.99	-1.77	2.85	.82
Babies fed with maternal milk grow up healthier than babies fed with artificial milk	1–5	3.29	1.29	-0.29	-0.90	.70
Breast milk is the ideal food for babies	1–5	4.65	0.67	-1.80	2.25	.89
Mother's milk is easier to digest than artificial milk	1–5	4.14	0.92	-0.60	-0.61	.61
Mother's milk is better than artificial milk	1–5	4.49	0.81	-1.60	2.31	.84
Are you breastfeeding your baby?	0–1	0.82	0.38	1.69	0.85	na
I’m planning to continue breastfeeding my baby for the next few months	1–5	4.70	0.69	-3.04	11.11	.90
My partner and family motivate me and support me to continue breastfeeding	1–5	4.26	1.03	-1.29	0.77	.68
Feeling good and satisfied motivates me to continue breastfeeding	1–5	4.53	0.82	-2.21	5.52	.84
Keeping the baby healthy is a motivation to continue breastfeeding	1–5	4.89	0.36	-3.38	11.59	.75
I offered my baby artificial milk before 4 months of age	1–5	1.95	1.39	1.31	0.26	.70
I have thought about giving up breastfeeding my baby	1–5	1.74	1.01	1.18	0.49	.90
I have had difficulties with breastfeeding due to the small amount of milk	1–5	1.64	1.17	1.82	2.25	.81
I have had difficulties with breastfeeding due to nipple problems	1–5	2.21	1.33	0.74	-0.69	.53
I have had difficulties with breastfeeding due to my work	1–5	1.70	1.17	1.42	0.74	.85
I have had difficulties with breastfeeding due to family problems	1–5	1.12	0.54	5.07	26.61	.94
I get comfortable to breastfeed my baby	1–5	4.42	0.66	-0.79	0.00	.79
I look for the correct position to breastfeed my baby	1–5	4.32	0.71	-0.94	1.51	
I know if my baby is drinking enough milk at the feed	1–5	3.61	1.00	-0.80	0.43	.66
I can breastfeed my baby without using artificial or powdered milk as a supplement	1–5	4.37	1.21	-1.88	2.25	.54
I'm sure that my baby latches onto the breast well during feeding	1–5	4.24	0.83	-1.21	2.07	.74
I can handle breastfeeding satisfactorily	1–5	4.04	1.01	-0.96	0.57	.91
Breastfeeding is a satisfactory experience for me	1–5	4.36	0.98	-1.69	2.60	.69
I can breastfeed my baby with one breast and then switch to the other	1–5	4.17	0.99	-1.15	0.76	.44

λ_ij_ saturation or factor loading

###### Breastfeeding practice

This is a scale made up of 4 first-order factors: motivation for breastfeeding; personal difficulties with breastfeeding; external difficulties with breastfeeding; and practical breastfeeding, explained by a global second order factor (c^2^=147.74, df=50, *p*< .001, c^2^/df=1.95, CFI=0.96, TLI=0.95, RMSEA (90%CI) = .09 (.07-.11), SRMR=0.07) (see Table 9). The internal consistency of the 12 items that make up the global factor was high (ordinal α = .85; ω=.76). The correlation with the PBSES scores is moderate (*r*=.41).

###### Self-confidence in breastfeeding

This is also a one-dimensional scale (c^2^=10.96, df= 9, *p*=.278, c^2^/df=1.21, CFI=0.99, TLI=0.99, RMSEA (90%CI) = .03 (.00-.08), SRMR=0.04) made up of 6 items (see Table 9) with high internal consistency (ordinal α= .82; ω=.77). The correlation with the PBSES scores is moderate (*r*=.43)

Scores of 22 or less in knowledge and self-efficacy for breastfeeding and equal to or less than 49 in practice are considered low.

##### Adaptation to role of mother

###### Parental self-efficacy

 This is a one-dimensional scale (χ^2^ = 270.72, df = 44, *p* < 0.001, χ^2^/df = 6.10, CFI = 0.99, TLI = 0.99, RMSEA (90%CI) = 0.12 (0.11-0.14), SRMR = 0.08) made up of 11 items (see Table 10) and very high internal consistency (ordinal α = 0.95; ω = 0.91). The correlation with the total score in the PSOC is moderate (rs = 0.60).

**Table 10:** Characteristics of the items that measure parental self-efficacy

	Min–max	M	SD	AI	Ku	λ_ij_
I am able to keep my baby entertained	1–5	4.08	0.79	-0.74	0.91	.75
I am able to feed my baby	1–5	4.88	0.43	-4.80	29.52	.74
I am able to bath my baby	1–5	4.73	0.74	-3.42	12.57	.54
I can calm my baby when she/he is crying	1–5	4.35	0.70	-0.95	1.29	.95
I am able to calm my baby when she/he is anxious	1–5	4.29	0.72	-0.81	0.78	.96
I can calm my baby when she/he cries continuously	1–5	4.19	0.77	-0.68	0.26	.92
I know when my baby is tired and needs to sleep	1–5	4.19	0.76	-0.69	0.38	.77
I can understand what my baby wants	1–5	3.86	0.69	-0.58	1.60	.80
I think my baby responds well to me when I talk and smile at her/him	1–5	4.45	0.74	-1.51	3.08	.84
I think my baby and I have good interaction	1–5	4.53	0.70	-1.56	2.73	.90
I can show affection to my baby	1–5	4.91	0.37	-6.23	51.16	.71

λ_ij_ saturation or factor loading

Scores of 52 or less would be indicative of low perceived self-efficacy.

###### Perceived social support

 This is a one-dimensional scale (χ^2^ = 54.6, df = 11, *p* < 0.001, χ^2^/df = 4.96, CFI = 0.99, TLI = 0.98, RMSEA (90%CI) = 0.13 (0.11-0.17), SRMR = 0.08) made up of 11 items (see Table 11) that present high internal consistency (ordinal α = 0.93; ω = 0.87). The correlation with the total score on the MOS-SSS is high (*r* = 0.80).

**Table 11:** Characteristics of the items that measure perceived social support

	Min–max	M	SD	AI	Ku	λ_ij_
There is a special person I can share sorrows and joys with	0–1	4.61	0.80	-2.33	5.51	.83
My family really try to help me	1–5	4.58	0.87	-2.21	4.54	.86
I get the help and emotional support I need from my family	1–5	4.36	0.98	-1.63	2.20	.94
I can talk about my problems with my family	1–5	4.37	0.99	-1.70	2.44	.92
I have friends I can share sorrows and joys with	1–5	4.32	0.96	-1.37	1.26	.73
There is a special person in my life who worries about my feelings	1–5	4.69	0.70	-2.52	6.30	.82

λ_ij_ saturation or factor loading

Scores of 24 or lower would be indicative of low perceived social support.

##### Mental Health

###### Self-image

 This is a scale made up of 8 items: 1 binary that acts as a key question and is used for seeing whether the woman is satisfied with her current physical appearance; and 7 politomic ones that evaluate the degree of discomfort that body dissatisfaction generates (see Table 12) (χ^2^ = 41.33, df = 14, *p* < 0.011, χ^2^/df = 2.95, CFI = 0.99, TLI = 0.99, RMSEA (90%CI) = 0.08 (0.05-0.11), SRMR = 0.06). Internal consistency is high (ordinal α = 0.90; ω = 0.87). The correlation of the scores with the body dissatisfaction scale of the EDI-3 used as the gold standard was strong (rs = 0.76).

**Table 12:** Characteristics of the items that measure self-image

	Min–max	M	SD	AI	Ku	λ_ij_
Are you happy with your current physical appearance?	0–1	0.42	0.49	0.31	-1.92	na
I avoid situations where people can see my body (e.g. pool/beach, bathrooms or changing rooms)	1–5	2.12	1.26	0.83	-0.46	.82
I worry about getting fat	1–5	2.82	1.37	0.16	-1.17	.87
I am afraid that my breasts will lose their shape or firmness	1–5	2.56	1.32	0.35	-1.02	.57
Seeing my body in the mirror makes me feel bad	1–5	2.36	1.28	0.64	-0.61	.92
I think I should go on a diet	1–5	2.92	1.51	0.04	-1.42	.80
I think I have lost most of the weight I gained during pregnancy	1–5	3.47	1.44	-0.52	-1.06	.52
I think my appearance is normal for a woman who has given birth recently	1–5	4.16	1.07	-1.18	0.65	.55

λ_ij_ saturation or factor loading, *na* not applicable

Scores of 22 or higher would indicate high body dissatisfaction.

###### Sleep problems

 This is a scale made up of 6 items: 1 binary that acts as a key question and is used to find out if the woman feels satisfied with her quality of sleep in general; and 5 politomic questions that evaluate the origin and type of difficulty (see Table 13) (χ^2^ = 9.53, df = 4, *p* = 0.049, χ^2^/df = 2.38, CFI = 0.97, TLI = 0.93, RMSEA (90%CI) = 0.10 (0.01-0.19), SRMR = 0.08). The internal consistency is acceptable (ordinal α = 0.73; ω = 0.67). The correlation of scores with ISI was moderate-high (rs = 0.61).

**Table 13:** Characteristics of items that measure sleep problems

	Min–max	M	SD	AI	Ku	λ_ij_
Are you satisfied with your quality of sleep?	0–1	0.52	0.50	0.09	-2.01	na
I wake up in the middle of the night	1–5	4.08	1.17	-1.28	0.81	.50
I have sleep problems due to child-care during the night	1–5	4.32	1.04	-1.53	1.55	.51
I have trouble sleeping due to anxiety related to the baby	1–5	2.13	1.21	0.82	-0.30	.64
I have sleep problems that leave me without energy throughout the day	1–5	3.31	1.13	-0.24	-0.56	.63
I have trouble getting to sleep	1–5	2.34	1.25	0.64	-0.55	.73

λ_ij_ saturation or factor loading, *na* not applicable

Scores of 19 or higher would indicate dissatisfaction with sleep quality.

###### Depression

 This is a one-dimensional scale made up of 6 items (see Table 14) (χ^2^ = 9.21, df = 9, *p* = 0.417, χ^2^/df = 1.01, CFI = 0.99, TLI = 0.99, RMSEA (90%CI) = 0.01 (0.00-0.07), SRMR = 0.05). The internal consistency is very high (ordinal α = 0.89; ω = 0.83). The correlation of scores with the EDPS is very high (rs = 0.82).

**Table 14:** Characteristics of the items that measure depression

	Min–max	M	SD	As	Cu	λ_ij_
I have felt lonely	1–5	2.11	1.13	0.65	-0.54	.75
I have cried a lot for no reason	1–5	2.05	1.06	0.67	-0.44	.73
I haven’t been able to concentrate on anything	1–5	2.16	1.10	0.63	-0.43	.82
I have felt as if I wasn’t myself	1–5	2.07	1.16	0.81	-0.34	.82
I have felt like a failure as a mother	1–5	1.77	0.98	1.15	0.72	.76
I have started thinking that I would be better off dead	1–5	1.06	0.33	5.88	37.52	.78

λ_ij_ saturation or factor loading

Scores of 14 or higher would indicate the presence of possible mood disorders.

## Discussion

When it comes to care of a woman during the postpartum period, as we mentioned in the introduction, it is of special importance to identify and measure not only serious morbidity, but also any other condition that prevents her from progressing in her recovery and adaptation to motherhood or that can affect her well-being in some way [10]. In this task, it is essential not only to take into account the results reported by patients or users of the health service, for example using PROs [57], but also that the measurement instruments enable the woman herself to obtain useful information and make decisions about her own health and that of her family. Likewise, in the selection of these instruments, in addition to demonstrating good metric characteristics and documentation for their interpretation [58–60], efficiency at the time of collecting the information is a quality that we must not overlook: if there is the option for selecting a short questionnaire, it should not be necessary to submit the patients to endless batteries of questions that gather identical information.

In line with this, given the lack of comprehensive, specific evaluation instruments with proven metric quality adapted to our environment, a digital instrument or dossier of scales made up of 99 items was developed, which evaluates 6 essential aspects for good psychosocial adjustment and successful coping mechanisms during the postpartum period. These aspects are in perfect harmony with the proposal of self-reported measurements by patients (PROs) that ICHOM made for the collection of health and well-being data in the postpartum period with a focus on the outcomes that matter most to patients [11]. It should be noted that it is a multidimensional self-evaluation instrument which covers the 6 priority areas proposed by the ICHOM and has several advantages: the first is that it can be used as a single measure of global or partial evaluation of the mentioned areas, making use of one or more sections separately; the second is that it is short, as in only 20 min it evaluates the six areas, which have been compared to gold standards equal or similar to those contained in the measurement tools proposed by ICHOM; the third is that it has been conceived and validated as a self-evaluation instrument by women, so it is useful for making shared decisions with the appropriate professional; and the fourth is that it is designed to be used in the EMAeHealth app, which was created through a collaborative research process [33], with the participation of puerperal women, professionals, managers and researchers. This app can easily be linked to the patient’s clinical e-records.

We believe that, if used correctly, it will be an instrument that will permit the collection of useful data for professionals related to postpartum care (e.g. gynaecologists, midwives, nurses and physiotherapists) and other health professionals, but above all that it will be useful for the woman herself, since it will allow for the exploration of her physical, social, emotional and sexual sphere in a short space of time, making it easy for her to make informed decisions about her health during the 12 months following delivery.

## Limitations

The ICHOM working groups understand that their function is not to design new measures of results, but to agree on a minimum set of well-validated measures, including the measures reported by patients, that everyone should use [61]. The purpose is to incorporate standard sets in patients’ medical e-records, to be able to make comparisons at different levels. Currently, our instrument has been developed in and for our health, social or cultural context; therefore, it would not be usable without previous adaptation and validation in a different environment. However, this fact is not something inherent to our tool but affects all psychometric measurement instruments; in fact, some recommend deepening evaluative research on the contribution of PRO instruments to a comparison between providers [62] which would contribute to the comparability of results. It might even be thought that, because it is a context-specific tool, it may have greater potential for use as a measurement tool in the clinical environment and in research implementation [63].

On the other hand, the fact that part of the sample has been selected by means of snowball sampling could increase the representation of more proactive women with a higher level of education. However, measures have been taken to avoid introducing bias: the overwhelming majority of the women were selected by 25 midwives belonging to public health centres located in various population areas, both rural and urban, and of different socioeconomic and social characteristics. Given that the women belonging to the snowball sample were referred by these same women, we can assume that they will come from equally varied backgrounds. In light of the sociodemographic data, it can be said that the women in our study are representative of the study population.

Finally, there is lack of evidence regarding the temporal stability of the scores. Information will probably be obtained on this aspect in future studies.

## Conclusions

This digital tool for measuring the priority health issues for women during the postpartum period, which is adapted to the cultural and health environment where it was designed (public primary gynaecological care), and which is in Spanish and has good psychometric properties, is considered useful and accessible for women and professionals.

The psychometric quality of the EMA-postpartum instrument, together with the advantages it presents in terms of format and length, would justify its consideration by the ICHOM as a possible PROM tool, within the Standard Set of Outcome Measures for Pregnancy and Childbirth, for the Health and Welfare section. However, a previous psychometric analysis of the properties of the scores derived from EMA-postpartum in other populations of pregnant women is recommended.

## Data Availability

The data sets generated and/or analyzed during the current study are not yet publicly available as they are still being processed by the research team for further publication, but can be made available from the corresponding author upon reasonable request.

## Abbreviations

AI: Asymmetry index
CFA: Confirmatory factor analysis
CFI: Comparative fit index
COSMIN: Consensus-based standards for the selection of health measurement instruments
DF: Degrees of freedom
DWLS: Diagonally weighted least squares
EDI-3: Eating disorder inventory-3
EFA: Exploratory factor analysis
EPDS: Edinburgh postnatal depression scale
ICIQ: International consultation on incontinence questionnaire short-form
ICHOM: International consortium for health outcomes measurement
K: Cohen’s kappa coefficient
Ku: Kurtosis index
LL: Lower median limit
M: Arithmetic mean
Max: Maximum
ME: Maternal education
Min: Minimum
MOS-SSS: Medical outcomes study-social support survey
Ordinal α: Ordinal alpha
PBSES: Prenatal breast-feeding self-efficacy scale
PSOC: Parental sense of competence
RMSEA: Root mean square error of approximation
SD: Standard deviation
SF-12 v2: Short form 12 items health survey version 2
SFQ: Sexual function questionnaire
SRMR: Standardized root mean square residual
TLI: Tucker-lewis index
UL: Upper median limit
λij: Standardized factorial weight
95% CI: Confidence interval of 95%
ω: McDonald’s Omega
χ^2^: Chi-squared

## References

[1] Sacks E, Langlois ÉV (2016). Postnatal care: increasing coverage, equity, and quality. Lancet Glob Health.

[2] WHO. Technical Consultation on Postpartum and Postnatal Care. Geneva: World Health Organization; 2010. PMID: 26269861. 26269861

[3] Daly D, Higgins A, Hannon S, O’Malley D, Wuytack F, Moran P, Cusack C, Begley C (2022). Trajectories of postpartum recovery: what is known and not known. Clin Obstet Gynecol.

[4] Grupo de trabajo de la Guía de práctica clínica de atención en el embarazo y puerperio. Guía de práctica clínica de atención en el embarazo y puerperio. Ministerio de Sanidad, Servicios Sociales e Igualdad. Agencia de Evaluación de Tecnologías Sanitarias de Andalicía; 2014. Guías de Práctica Clínica en el SNS: AETSA 2011/10.

[5] Lambermon FJ, van Duijnhoven NTL, Braat DDM, Kremer JAM, Dedding CWM (2020). The unintended consequences of client-centred flexible planning in home-based postpartum care: a shift in care workers’ tasks and responsibilities. Midwifery.

[6] Paz Pascual C, ArtietaPinedo I, Grandes G, Espinosa Cifuentes M, GamindeInda I, Payo Gordon J (2016). Necesidades percibidas por las mujeres respecto a su maternidad. Estudio cualitativo para el rediseño de la educación maternal [Perceived needs of women regarding maternity. Qualitative study to redesign maternal education]. Aten Primaria.

[7] Verbiest S, Tully K, Simpson M, Stuebe A (2018). Elevating mothers' voices: recommendations for improved patient-centered postpartum. J Behav Med.

[8] Verbiest S, Bonzon E, Handler A (2016). postpartum health and wellness: a call for quality woman-centered care. Matern Child Health J.

[9] Paz-Pascual C, Artieta-Pinedo I, Grandes G, ema.QGroup (2019). Consensus on priorities in maternal education: results of Delphi and nominal group technique approaches. BMC Pregnancy Childbirth.

[10] Jolivet RR, Moran AC, O'Connor M, Chou D, Bhardwaj N, Newby H, Requejo J, Schaaf M, Say L, Langer A (2018). Ending preventable maternal mortality: phase II of a multi-step process to develop a monitoring framework, 2016–2030. BMC Pregnancy Childbirth.

[11] Nijagal MA, Wissig S, Stowell C, Olson E, Amer-Wahlin I, Bonsel G, Brooks A, Coleman M, Devi Karalasingam S, Duffy JMN, Flanagan T, Gebhardt S, Greene ME, Groenendaal FR, Jeganathan JR, Kowaliw T, Lamain-de-Ruiter M, Main E, Owens M, Petersen R, Reiss I, Sakala C, Speciale AM, Thompson R, Okunade O, Franx A (2018). Standardized outcome measures for pregnancy and childbirth, an ICHOM proposal. BMC Health Serv Res..

[12] Moraes GP, Lorenzo L, Pontes GA, Montenegro MC, Cantilino A (2017). Screening and diagnosing postpartum depression: when and how?. Trends Psychiatry Psychother.

[13] Zubaran C, Schumacher M, Roxo MR, Foresti K (2010). Screening tools for postpartum depression: validity and cultural dimensions. Afr J Psychiatry (Johannesbg).

[14] O'Connor E, Rossom RC, Henninger M, Groom HC, Burda BU (2016). Primary care screening for and treatment of depression in pregnant and postpartum women: evidence report and systematic review for the US preventive services task force. JAMA.

[15] Petrozzi A, Gagliardi L (2013). Anxious and depressive components of Edinburgh Postnatal Depression Scale in maternal postpartum psychological problems. J Perinat Med.

[16] Brunton RJ, Dryer R, Saliba A, Kohlhoff J (2015). Pregnancy anxiety: a systematic review of current scales. J Affect Disord.

[17] Gard E, Lyman A, Garg H (2022). Perinatal incontinence assessment tools: a psychometric evaluation and scoping review. J Womens Health (Larchmt).

[18] Ngo LTH, Chou HF, Gau ML, Liu CY (2019). Breastfeeding self-efficacy and related factors in postpartum Vietnamese women. Midwifery.

[19] Ho YJ, McGrath JM (2010). A review of the psychometric properties of breastfeeding assessment tools. J Obstet Gynecol Neonatal Nurs.

[20] Piñeiro-Albero RM, Ramos-Pichardo JD, Oliver-Roig A, Velandrino-Nicolás A, Richart-Martínez M, García-de-León-González R, Wells KJ (2013). The Spanish version of the prenatal breast-feeding self-efficacy scale: reliability and validity assessment. Int J Nurs Stud.

[21] Mahumud RA, Ali N, Sheikh N, Akram R, Alam K, Gow J, Sarker AR, Sultana M (2019). Measuring perinatal and postpartum quality of life of women and associated factors in semi-urban Bangladesh. Qual Life Res.

[22] Brekke M, Berg RC, Amro A, Glavin K, Haugland T (2022). Quality of Life instruments and their psychometric properties for use in parents during pregnancy and the postpartum period: a systematic scoping review. Health Qual Life Outcomes.

[23] Evans K, Fraser H, Uthman O, Osokogu O, Johnson S, Al-Khudairy L (2022). The effect of mode of delivery on health-related quality-of-life in mothers: a systematic review and meta-analysis. BMC Pregnancy Childbirth.

[24] Mokhtaryan-Gilani T, Kariman N, Nia HS, Doulabi MA, Nasiri M (2022). The Maternal Postpartum Quality of Life Instrument (MPQOL-I): development and psychometric evaluation in an exploratory sequential mixed-method study. BMC Pregnancy Childbirth.

[25] Badr HA, Zauszniewski JA (2017). Meta-analysis of the predictive factors of postpartum fatigue. Appl Nurs Res.

[26] Sultan P, Ando K, Sultan E, Hawkins J, Blake L, Barwick F, Kawai M, Carvalho B (2021). A systematic review of patient-reported outcome measures used to assess sleep in postpartum women using Consensus Based Standards for the Selection of Health Measurement Instruments (COSMIN) guidelines. Sleep.

[27] López-Lapeyrere C, Serna-Gómez N, Hernández-López AB, Pérez-García MF, Tejeda-Esteban A, Solís-Muñoz M (2018). The development and validation of a new postpartum sexual function and dyspareunia assessment tool: the carol scale. Midwifery.

[28] Denis A, Callahan S, Bouvard M (2015). Evaluation of the French version of the multidimensional scale of perceived social support during the postpartum period. Matern Child Health J.

[29] Busonera A, Cataudella S, Lampis J, Tommasi M, Zavattini GC (2017). Psychometric properties of the Postpartum Bonding Questionnaire and correlates of mother-infant bonding impairment in Italian new mothers. Midwifery.

[30] Wittkowski A, Wieck A, Mann S (2007). An evaluation of two bonding questionnaires: a comparison of the Mother-to-Infant Bonding Scale with the Postpartum Bonding Questionnaire in a sample of primiparous mothers. Arch Womens Ment Health.

[31] O’Byrne LJ, Bodunde EO, Maher GM, Khashan AS, Greene RM, Browne JP, Mccarthy FP (2022). Patient reported outcome measures evaluating postpartum maternal health and wellbeing: a systematic review and evaluation of measurement properties. Am J Obstet Gynecol MFM.

[32] Sultan P, Sharawi N, Blake L, Ando K, Sultan E, Aghaeepour N, Carvalho B, Sadana N (2021). Use of patient-reported outcome measures to assess outpatient postpartum recovery: a systematic review. JAMA Netw Open.

[33] Artieta-Pinedo I, Paz-Pascual C, Bully P, Espinosa M, EmaQGroup (2021). Design of the Maternal Website EMAeHealth that supports decision-making during pregnancy and in the postpartum period: collaborative action research study. JMIR Form Res.

[34] Paz-Pascual C, Artieta-Pinedo I, Espinosa M, Bully P, ema-Q Group (2020). Development of two instruments for assessing maternity health needs: protocol of a clinimetric study. BMC Pregnancy Childbirth.

[35] Mokkink LB, Prinsen C, Patrick DL, Alonso J, Bouter LM, de Vet HC (2018). COSMIN methodology for systematic reviews of patient-reported outcome measures (PROMs). User manual.

[36] Kyriazos TA (2018). Applied psychometrics: sample size and sample power considerations in factor analysis (EFA, CFA) and SEM in general. Psychology.

[37] Singh K, Junnarkar M, Kaur J (2016). Measures of positive psychology.

[38] Hu LT, Bentler PM (1999). Cutoff criteria for fit indexes in covariance structure analysis: Conventional criteria versus new alternatives. Struct Equ Model.

[39] Kenny DA, Kaniskan B, McCoach DB (2015). The performance of RMSEA in models with small degrees of freedom. Sociol Methods Res.

[40] Sánchez-García JC, Aguilar-Cordero M, Menor-Rodríguez M, Paucar Sánchez AM, Rodríguez-Blanque R (2019). Influencia del ejercicio físico en la evolución del peso gestacional y posparto Ensayo clínico aleatorizado. Nutr Hospital.

[41] Daly D, Moran P, Wuytack F, Hannon S, Hannon K, Martin Y, Peoples M, Begley C, Newnham E (2022). The maternal health-related issues that matter most to women in Ireland as they transition to motherhood - A qualitative study. Women Birth.

[42] Afshari P, Dabagh F, Iravani M, Abedi P (2017). Comparison of pelvic floor muscle strength in nulliparous women and those with normal vaginal delivery and cesarean section. Int Urogynecol J.

[43] O’Malley D, Higgins A, Smith V. Exploring the complexities of postpartum sexual health. Curr Sex Health Rep. 2022;13:128–35. 10.1007/s11930-021-00315-6.

[44] Triviño-Juárez JM, Romero-Ayuso D, Nieto-Pereda B, Forjaz MJ, Oliver-Barrecheguren C, Mellizo-Díaz S, Plá-Mestre R (2018). Resumption of intercourse, self-reported decline in sexual intercourse and dyspareunia in women by mode of birth: a prospective follow-up study. J Adv Nurs.

[45] Tully KP, Stuebe AM, Verbiest SB (2017). The fourth trimester: a critical transition period with unmet maternal health needs. Am J Obstet Gynecol.

[46] Lawson A, Murphy KE, Sloan E, Uleryk E, Dalfen A (2015). The relationship between sleep and postpartum mental disorders: a systematic review. J Affect Disord.

[47] Martín-Gómez C, Moreno-Peral P, Bellón JA, Conejo-Cerón S, Campos-Paino H, Gómez-Gómez I, Motrico E. Effectiveness of psychological interventions in preventing postpartum depression in non-depressed women: a systematic review and meta-analysis of randomized controlled trials. Psychol Med. 2022;52(6):1001–13. 10.1017/S0033291722000071. 10.1017/S0033291722000071 35257650

[48] Schmidt S, Vilagut G, Garin O, Cunillera O, Tresserras R, Brugulat P, Mompart A, Medina A, Ferrer M, Alonso J (2012). Normas de referencia para el Cuestionario de Salud SF-12 versión 2 basadas en población general de Cataluña. Med Clin.

[49] Espuña Pons M, Rebollo Alvarez P, Puig Clota M (2004). Validación de la versión española del International Consultation on Incontinence Questionnaire-Short Form. Un cuestionario para evaluar la incontinencia urinaria. Med Clin (Barc).

[50] Sánchez F, Conchillo MP, Valls JB, Llorens OG, Vicentee JA, de Las Mulas ACM (2004). Diseño y validación del cuestionario de Función Sexual de la Mujer (FSM). Atención primaria.

[51] Piñeiro-Albero RM, Ramos-Pichardo JD, Oliver-Roig A, Velandrino-Nicolás A, Richart-Martínez M, García-de-León-González R (2013). The Spanish version of the prenatal breast-feeding self-efficacy scale: reliability and validity assessment. Nurs Stud.

[52] Oltra‐Benavent P, Cano‐Climent A, Oliver‐Roig A, Cabrero‐García J, Richart‐Martínez M. Spanish version of the parenting sense of competence scale: evidence of reliability and validity. Child Fam Soc Work. 2020;25(2):373–83. 10.1111/cfs.12693.

[53] Costa Requena G, Salamero M, Gil F (2007). Validación del cuestionario MOS-SSS de apoyo social en pacientes con cáncer. Med Clin.

[54] Bully P, Elosua P (2011). Changes in body dissatisfaction relative to gender and age: the modulating character of BMI. Span J Psychol.

[55] Fernandez-Mendoza J, Rodriguez-Muñoz A, Vela-Bueno A, Olavarrieta-Bernardino S, Calhoun SL, Bixler EO, Vgontzas AN (2012). The Spanish version of the insomnia severity index: a confirmatory factor analysis. Sleep Med.

[56] Gutierrez-Zotes A, Gallardo-Pujol D, Labad J, Martín-Santos R, García-Esteve L, Gelabert E, Jover M, Guillamat R, Mayoral F, Gornemann I, Canellas F, Gratacós M, Guitart M, Roca M, Costas J, Ivorra JL, Navinés R, de Diego Y, Vilella E, Sanjuan J (2018). Factor Structure of the Spanish version of the Edinburgh postnatal depression scale. Actas Esp Psiquiatr.

[57] FDA. Guidance for Industry. Patient-Reported Outcome Measures: Use in Medical Product Development to Support Labeling Claims. Food and Drug Administration (FDA). Disponible en: http://www.fda.gov/downloads/Drugs/Guidances/UCM193282.pdf. Access on Feb 2023.

[58] Reeve BB, Wyrwich KW, Wu AW (2013). ISOQOL recommends minimum standards for patient-reported outcome measures used in patient-centered outcomes and comparative effectiveness research. Qual Life Res.

[59] Mokkink LB, Prinsen CA, Bouter LM, Vet HC, Terwee CB (2016). The COnsensus-based Standards for the selection of health Measurement INstruments (COSMIN) and how to select an outcome measurement instrument. Braz J Phys Ther.

[60] Mokkink LB, Terwee CB, Patrick DL, Alonso J, Stratford PW, Knol DL (2010). The COSMIN checklist for assessing the methodological quality of studies on measurement properties of health status measurement instruments: an international Delphi study. Qual Life Res.

[61] Porter ME, Larsson S, Lee TH (2016). Standardizing patient outcomes measurement. N Engl J Med.

[62] https://fundaciongasparcasal.org/wp-content/uploads/2021/02/Monografia-4-Politica_Resultados-reportados-por-los-pacientes.pdf. Acceso en Feb 2023. ISBN: 978–84–16732–60–9 Depósito Legal: M-7693–2017

[63] Kaplan HC, Walsh KE (2022). Context in implementation science. Pediatrics.

